# Patient and Tumor Factors on Overall Survival in Spindle Cell Melanoma

**DOI:** 10.7759/cureus.74247

**Published:** 2024-11-22

**Authors:** Xinxin Wu, Peter T Silberstein

**Affiliations:** 1 College of Medicine, Creighton University School of Medicine, Omaha, USA; 2 Oncology, Creighton University School of Medicine, Omaha, USA

**Keywords:** demographics, health disparity, overall survival, skin cancer, spindle cell melanoma

## Abstract

Introduction: Spindle cell melanoma (SCM) is a rare melanoma subtype characterized histologically by atypical, spindled, malignant melanocytes. SCM often presents in older patients and lacks typical cytologic markers, complicating diagnosis and frequently leading to advanced disease upon presentation. While some studies have documented SCM incidence, none have thoroughly examined the demographic, socioeconomic, and treatment factors influencing survival outcomes.

Objective: This study aims to investigate how demographic and clinical factors affect overall survival outcomes in SCM, providing updated data on demographic, socioeconomic, and tumor-related characteristics and treatment patterns.

Methods: This retrospective cohort study analyzed histologically confirmed spindle cell melanoma and mixed spindle cell melanoma cases diagnosed between 2004 and 2021. Patient demographic, socioeconomic, and clinical factors were collected, and multivariate Cox regression analysis was performed to determine the effects on overall survival.

Results: Among 9,210 SCM cases, most were White patients (97.5%), male (65.2%), and located in metropolitan areas (85.7%). Males were more likely to receive treatment at academic centers and have higher comorbidity scores. The average age at diagnosis was 67.3 years (SD±15.1). SCM in head and neck regions showed a worse prognosis compared to extremity melanomas. Surgical intervention, received by 84.9% of patients, was associated with improved overall survival. The minority of patients who received radiation and chemotherapy (13.2% and 3.3%, respectively) were linked to increased mortality risk.

Conclusion: Age, sex, insurance status, treatment facility type, tumor location, and stage significantly influence SCM survival outcomes. The findings suggest that SCM’s prognosis is closely tied to demographic and clinical characteristics, similar to other melanoma subtypes, supporting standard treatment protocols with surgical intervention as the primary approach. This large-scale study leverages comprehensive NCDB data, contributing to the literature gap in SCM’s treatment and management.

## Introduction

Spindle cell melanoma (SCM) is a rare morphological subtype of melanoma, characterized histologically by atypical, spindled, malignant melanocytes [1]. Retrospective studies have shown SCM, with either a pure or mixed spindle cell component, to occur in a higher age group compared to melanoma, lack classical cytological features of melanoma, and have unreliable markers for staining and immunoreactivity [2]. SCM often manifests as a broad metastatic illness, is frequently amelanotic, and can arise nearly anywhere on the body, making the diagnosis challenging. Due to the frequent delay in diagnosing SCM, patients often present with advanced-stage disease characterized by extensive metastasis, which contributes to poor treatment outcomes [3].

While several studies have analyzed the incidence of SCM, there is a gap in understanding the interplay between sociodemographic and treatment patterns in survival outcomes. For instance, previous studies on rare melanomas identified disparities in outcomes based on race, sex, age, and tumor location, but other socioeconomic, tumor, or treatment factors were not assessed. Non-Hispanic White patients and female sex had a better melanoma-specific survival rate, whereas older age had a worse melanoma-specific survival [4-8]. Some studies showed that head and neck melanomas had a worse prognosis, with the scalp being the worst prognostic factor [9]. Other studies have shown that head/neck and trunk melanomas were more likely than limb melanomas to metastasize and have worse outcomes [10].

Given the rarity of SCM, literature on the epidemiology of SCM has not yet been fully explored. The incidence has been documented in a population-based surveillance, epidemiology, and end results (SEER) study of 4,761 patients diagnosed with SCM with males, Caucasian race, and age in the 6-8th decade of life more likely to be associated with diagnosis [5]. Similar to melanoma, age, sex, ethnicity, and tumor location serve as prognostic factors [4-8]. However, SEER contains limited data on socioeconomic variables and hospital-based data, which are critical in understanding healthcare access and disparities [11]. The National Cancer Database (NCDB) is a joint project of the Commission on Cancer of the American College of Surgeons and the American Cancer Society Nationwide Oncology Outcomes Database for more than 1,500 commission-accredited cancer programs in the United States and Puerto Rico. The NCDB provides critical data that addresses gaps in the existing literature, particularly in areas where previous studies have lacked comprehensive demographic, treatment, and outcome information. Therefore, we aim to identify these characteristics, treatments, and survival outcomes associated with spindle cell melanoma (SCM) using a large nationwide database, leveraging a substantial sample size to assess the impact of various factors on survival.

Our preliminary studies indicated that males were more likely than females to be diagnosed at an older age, receive treatment at an academic center compared to a non-academic center, and have a higher co-morbidity index [12]. In this study, we continue the investigation to evaluate the impact of not only sex but also race, socioeconomic factors, histologic cell pathology, and treatment variables on overall survival outcomes in SCM. This comprehensive approach aims to provide a deeper understanding of how these factors interact and influence prognosis.

## Materials and methods

This retrospective cohort study analyzed patients diagnosed with histologically confirmed SCM between 2004 and 2021 sourced from the NCDB with ICD-O-3 histology code 8772 or 8770 for spindle cell melanoma, not otherwise specified (NOS) and mixed epithelial and spindle cell melanoma, respectively. The NCDB, jointly sponsored by the American College of Surgeons and the American Cancer Society, compiles information from over 1,500 commissions on cancer-accredited facilities and covers more than 70% of new cancer diagnoses in the United States and Puerto Rico. De-identified patient data was granted through the participant user data files program. 

The demographic factors analyzed include age, sex, race, Hispanic ethnicity, insurance type, facility type, population density, income, education level, Charlson-Deyo comorbidity score, tumor characteristics (location, stage, histology, and behavior), and treatments received (surgical, radiation, immunotherapy, and chemotherapy). Cases categorized as 90 years or older without nominal variables were excluded from the analysis.

Race was categorized into three groups due to limited data, which are White patients, Black patients, and Other. The "Other" category included subgroups of American Indian patients, Aleutian or Eskimo patients, Chinese patients, Japanese patients, Filipino patients, Hawaiian patients, Korean patients, Vietnamese patients, Kampuchean patients, Asian Indian or Pakistani NOS patients, Asian Indian patients, Micronesian NOS patients, other Asian patients, Asian NOS patients, Oriental NOS patients, Pacific Islander NOS patients, other and unknown patients. Hispanic origin was determined by self-reported Spanish or Hispanic heritage.

Insurance status was determined by the primary payor at diagnosis and categorized into five groups: no insurance, private insurance, Medicare, Medicaid, and other government insurance. Treatment facilities were categorized into Community Cancer Program, Comprehensive Community Cancer Program, Academic/Research Program, and Integrated Network Cancer Program by facility, program structure, services provided, and the number of cases accessed each year. Population density encompassed metropolitan, urban, and rural designations using the typology published by the United States Department of Agriculture (USDA) Economic Research Service. The income quartile was determined by median household income determined by the 2016 to 2020 census in the patient's zip code of residence at the time of diagnosis. Education was measured into quartiles by the percentage of residents in the patient's zip code who did not have a high school degree based on the 2016 to 2020 census. Comorbidities were assessed using the Charlson-Deyo score, and patients were divided into groups with scores of 0, 1, 2, and ≥3. Tumor location was defined as the primary site of the tumor. The tumor stage was defined using the NCDB analytical stage criteria, which uses the pathologic stage group and the clinical stage group when the former is unavailable. Histology and behavior codes were reported to the NCDB using the International Classification of Disease for Oncology, Third Edition, and Morphology, respectively. All treatments were categorized dichotomously as either received or not received.

All analyses were conducted with SPSS version 29 (SPSS) version 29 (IBM Corp., Armonk, NY). Overall survival (OS) was assessed using Kaplan-Meier log-rank analyses. A Cox regression multivariate model was employed to identify independent prognostic factors in overall survival. All descriptive variables were included in the multivariable Cox model. Exclusion criteria included missing values. Statistical significance for all analyses was set at p<0.05. Creighton University Institutional Review Board (IRB) has determined that this project, under submission number 2003680-01, does not involve human subjects under 45 CFR46.102(e), and an IRB review was not required.

## Results

The NCDB identified 9,210 patients, who were histologically defined as mixed epithelial and spindle cell melanoma or spindle cell melanoma, NOS (Table 1). A minority of cases had mixed histological features (14%).

**Table 1:** Number of cases by International Classification of Disease for Oncology, Third Edition (ICD-O-3) Histologic Code.

ICD-O-3 code	Histology	N	Valid percent
8770	Mixed epithelial and spindle cell melanoma	1291	14
8772	Spindle cell melanoma, NOS	7919	86
Total		9210	

Descriptive variables are listed in Table 2. The average age of an individual diagnosed with SCM was 67.3 (SD 15.1) months. Males had a predominant frequency compared to females (65.2% vs 34.8%). The vast majority of patients self-reported their race as White patients (97.5%), with less than one percent self-reporting as Black patients (0.8%). Only 4.9% of patients were of Hispanic origin. Most patients had Medicare insurance (55.9%), followed by private insurance (37.8%), Medicaid insurance (2.8%), no insurance (2.2%), or other government insurance (1.3%). Almost half of the patients were treated at an academic/research center (46.8%) and in metropolitan areas (85.7%). The highest income quartile was over-represented in our sample at 44.6%. The top two education quartiles had greater representation than the bottom two, with 32.1% in the second-highest quartile and 29.0% in the highest quartile. Patients diagnosed with SCM were generally in good health, with over 90% of the sample having a Charlson-Deyo score of 0 or 1. Primary tumor locations varied, with 39.8% of patients having tumors on the head or neck, 34.0% on the extremities, and 17.9% on the trunk. The majority of cases were categorized as Stage II (48.3%), followed by Stage I (27.6%), Stage III (14.1%), Stage IV (9.3%), and Stage 0 (0.6%). Most cases had invasive tumor behavior, accounting for 99.8% of patients, while in-situ tumors comprised only 0.2% of cases. Surgery was the most popular treatment of choice. Surgery was performed in 84.9% of cases, with 15.1% of patients not undergoing any surgical intervention. Radiation therapy was not utilized in 86.8% of cases, with 13.2% of patients receiving treatment. The use of immunotherapy was limited to 9.7%. Chemotherapy was the least commonly used treatment modality, with only 3.3% undergoing chemotherapy. Among the 8,768 patients with known survival data, the mean survival time was 124.3 months (SE=1.2), while the median survival time was 120.0 months (SE=3.1).

**Table 2:** Demographic/socioeconomic, tumor-related, and treatment-related breakdown of SCM.

N=9210		Frequency (n)	Valid percent (%)	Mean (if applicable)
Age (years)	Mean (SD)			67.3 (15.1)
Sex	Male	6006	65.2	
	Female	3204	34.8	
Race	White patients	8981	97.5	
	Black patients	70	0.8	
	Other	159	1.7	
Hispanic origin	Non-Spanish, Non-Hispanic patients	8759	95.1	
	Hispanic patients	448	4.9	
Insurance status	Not insured	196	2.2	
	Private insurance	3437	37.8	
	Medicaid	256	2.8	
	Medicare	5080	55.9	
	Other government	114	1.3	
Treatment facility	Community cancer program	337	3.9	
	Comprehensive community cancer program	2621	30.1	
	Academic/research program	4077	46.8	
	Integrated network cancer program	1673	19.2	
Urban/rural (2020)	Metropolitan	7892	85.7	
	Urban	1248	13.6	
	Rural	70	0.8	
Median household income (2020)		873	11.0	
	$57,857-$74,062	1513	19.1	
	$46,277-$57,856	2002	25.3	
	$74,063	3534	44.6	
Percent without a high school degree	>=15.3%	1105	13.9	
	9.1%-5.2%	1981	24.9	
	5.0%-9.0%	2552	32.1	
	<=5.0%	2304	29.0	
Charlson–Deyo score	0	7488	81.3	
	1	1223	13.3	
	2	317	3.4	
	>=3	182	2.0	
Primary tumor location	Skin of the head or neck	3639	39.8	
	Skin of trunk	1636	17.9	
	Skin of the extremities	3101	34.0	
	Overlapping lesion of skin or NOS	756	8.3	
Stage	0	51	0.6	
	I	2254	27.6	
	II	3939	48.3	
	III	1153	14.1	
	IV	762	9.3	
Histology	Mixed epithelial and spindle cell melanoma	1291	14.0	
	Spindle cell melanoma, NOS	7919	86.0	
Behavior	In-situ and synonymous with in-situ	15	.2	
	Invasive	9195	99.8	
Surgery	Not performed	1389	15.1	
	Performed	7820	84.9	
Radiation	Not performed	7996	86.8	
	Performed	1211	13.2	
Immunotherapy	Not performed	8274	90.3	
	Performed	887	9.7	
Chemotherapy	Not performed	8829	96.7	
	Performed	300	3.3	
Survival (n=8,768; months)	Mean (SE)			124.3 (1.2)
	Median (SE)			120.0 (3.1)

Multivariate analysis, outlined in Table 3, showed that overall survival was significantly associated with age, sex, race, Hispanic origin, type of insurance, treatment facility type, comorbidity status, and the use of surgical, radiation, or chemotherapy treatments.

**Table 3:** Multivariate analysis of overall survival (cox-regression).

N = 9210		Hazard ratio	95% Confidence interval	p-value
Age		1.061	1.058-1.065	<0.001
Sex	Male	reference		
	Female	0.864	0.802-0.932	<0.001
Race	White patients	reference		
	Black patients	1.740	1.232 - 2.455	0.006
	Other00	0.929	0.708-1.221	0.599
Hispanic origin	Non-Spanish, Non-Hispanic patients	reference		
	Hispanic patients	1.159	0.998 - 1.346	0.056
Insurance status	Not insured	reference		
	Private insurance	0.611	0.475-0.787	<0.001
	Medicaid	1.162	0.848-1.594	0.350
	Medicare	0.638	0.494 - 0.824	<0.001
	Other government	0.557	0.356 - 0.870	0.010
Treatment facility	Community cancer program	reference		
	Comprehensive community cancer program	0.691	0.433-0.861	0.040
	Academic/research program	0.604	0.398-0.742	0.002
	Integrated network cancer program	0.573	0.369-0.683	<0.001
Urban/rural (2020)	Metropolitan	reference		
	Urban	0.964	0.871-1.068	0.488
	Rural	1.222	0.863-1.728	0.258
Median household income (2020)		reference		
	$57,857-$74,062	0.781	0.386 - 1.581	0.492
	$46,277-$57,856	0.714	0.354-1.440	0.347
	$74,063	0.683	0.340-1.375	0.286
Percent without a high school degree	>=15.3%	reference		
	9.1%-15.2%	1.900	0.934-3.864	0.076
	5.0%-9.0%	1.698	0.837-3.443	0.142
	<5.0%	1.665	0.823-3.369	0.156
Charlson–Deyo score	0	reference		
	1	1.132	1.033-1.240	0.008
	2	1.521	1.299-1.781	<0.001
	>=3	1.922	1.579-2.341	<0.001
Primary tumor location	Skin of the head or neck	reference		
	Skin of trunk	1.034	0.938-1.140	0.502
	Skin of the extremities	0.803	0.738-0.873	<0.001
	Overlapping lesion of skin or NOS	0.914	0.786-1.063	0.243
Stage	0	reference		
	I	0.667	0.391-1.138	0.137
	II	1.030	0.606-1.750	0.914
	III	1.501	0.878-2.565	0.138
	IV	3.278	1.908-5.635	<0.001
Histology	Mixed epithelial and spindle cell melanoma	reference		
	Spindle cell melanoma, NOS	1.024	0.920-1.140	0.664
Behavior	In-situ and synonymous with in-situ	reference		
	Invasive	1.044	0.405-2.688	0.930
Surgery	Not performed	reference		
	Performed	0.841	0.750-0.943	0.003
Radiation	Not performed	reference		
	Performed	1.257	1.146-1.379	<0.001
Immunotherapy	Not performed	reference		
	Performed	0.770	0.677-0.875	<0.001
Chemotherapy	Not performed	reference		
	Performed	1.604	1.370-1.877	<0.001

Increased age was linked to worse overall survival (HR:1.061, p<0.001). Female sex was associated with better overall survival compared to males (HR:0.864, p<0.001). Compared to White patients, Black patients had worse overall survival (HR:1.740, p=0.006). Compared to uninsured patients, those with private insurance (HR:0.611, p<0.001), Medicare (HR:0.638, p<0.001), and other government insurance (HR:0.557, p=0.010) had significantly better overall survival. Patients treated at Comprehensive Community Cancer Programs, Academic/Research Programs, and Integrated Network Cancer Programs all had significantly better overall survival compared to those treated at Community Cancer Programs (p<0.05). Compared to patients with a Charlson-Deyo score of 0, patients with a score of 1 had a hazard ratio of 1.132 (p=0.008), while those with a score of 2 had a hazard ratio of 1.521 (p<0.001), and those with a score of ≥3 had a hazard ratio of 1.922 (p<0.001), indicating that higher scores are associated with a greater risk of mortality. If the primary tumor location was the skin of the extremities, it was associated with better overall survival compared to lesions on the head or neck (HR:0.803, p<0.001). However, tumors located on the skin of the trunk, overlapping tumors, or sites NOS did not show a statistically significant difference in survival compared to those on the head or neck (p>0.05). Stage IV tumors were associated with a significantly higher hazard ratio for mortality (HR:3.278, p<0.001) compared to Stage 0. Patients who underwent surgical treatment had a significantly lower risk of mortality compared to those who did not have surgery (HR:0.841, p=0.003). Immunotherapy was also associated with a lower risk of mortality compared to patients who did not receive this treatment (HR:0.770, p<0.001). In contrast, patients who received radiation or chemotherapy had a significantly increased risk of mortality compared to those who did not undergo these treatments (HR:1.257 and 1.604, p<0.001, respectively). Population density, Hispanic origin, income, education, tumor histology, and tumor behavior did not demonstrate a statistically significant association with overall survival.

## Discussion

This study corroborates previous research indicating that older age, male sex, Black patients, comorbidity status, and location of the tumor on the head/neck are significant negative prognostic factors in SCM, paralleling other findings in overall melanoma and rare melanoma subtypes [4-10, 13]. These results emphasize the importance of these demographic and clinical variables in predicting outcomes, supporting their continued consideration in treatment planning and patient management. Our study also contributes to the existing literature by demonstrating a primary tumor location on the extremity, private or non-Medicaid government insurance, a facility that diagnoses over 500 cancer cases a year, and the use of surgical and immunotherapeutic treatments as significant positive prognostic factors [14]. Previous studies on chemotherapy in metastatic melanoma have yielded mixed results [15]. We demonstrate here that radiation and chemotherapy are associated with a higher hazard ratio. However, we must consider that these factors may also be influenced by tumor factors not limited to the chosen variable in this study; for instance, patients with more advanced diseases may be directed to different treatment facilities, present with distinct comorbidities, and receive varied treatment approaches, which could, in turn, impact survival outcomes. 

The average age of melanoma and previous median age for SCM in the USA is 66 years, slightly younger than the average age of patients in this study at 67.3 years [1, 16]. It is noteworthy that Medicare eligibility in the United States begins at age 65 [17]. This suggests that a significant proportion of patients within this cohort are likely enrolled in Medicare or are of an age where their financial situation may be stabilized, allowing them to access necessary medical assistance. The analysis reveals that those insured under Medicare, insurance, or other government insurance had a statistically significant survival advantage compared to those without insurance. Conversely, patients with Medicaid, who may represent a lower socioeconomic status or have more complex health needs, had no statistically significant difference in survival compared to those without insurance. Notably, our cohort includes patients pre and post-Medicaid expansion under the Affordable Care Act, which broadened Medicaid eligibility [18]. The data supports the assertion that insurance status is crucial in ensuring better overall survival, especially for patients not already facing socioeconomic barriers or healthcare disparity, such as those eligible for Medicaid. The issue is complex, as our study determined income and education are inconsequential in overall survival, suggesting possible underlying factors not explored here. 

Patients experienced better outcomes at academic/research and integrated network cancer programs or comprehensive programs than community programs, likely due to access to advanced treatments, clinical trials, multidisciplinary teams, and other advantages associated with universities or research institutions. Interestingly, despite having easier access to academic or integrated centers, living in a more populous area did not impact overall survival. 

The observed increase in mortality risk among patients who received radiation and chemotherapy may be attributed to the underlying characteristics of the tumors requiring these treatments. It is plausible that tumors necessitating radiation or chemotherapy are inherently more advanced, which could predispose these patients to poorer outcomes or be used palliatively [15]. Cases elected for surgery or immunotherapy could have less tumor burden or ulceration. Therefore, the higher risk of mortality in this cohort may not solely reflect the effects of the treatments but rather the nature of the tumors that warranted such interventions.

We propose that pure and mixed spindle cell histology do not significantly differ in overall survival outcomes, suggesting that both histological types may warrant similar treatment approaches. Surgical intervention remains the mainstay of treatment, similar to other cutaneous carcinomas [10, 19].

## Conclusions

In the largest study conducted to date, we analyze demographic, socioeconomic, tumor-related, and treatment patterns in patients with spindle cell melanomas. A key strength of our study is the use of comprehensive data from the NCDB, which, combined with the substantial sample size of melanoma cases, enhances the reliability of our findings. From this analysis, we conclude that SCM shares similar prognostic factors with melanoma, including age, sex, insurance status, treatment facility, comorbidities, primary site location, stage, and therapy, with possible outside factors, such as access to resources and healthcare disparity, playing a role. The current treatment patterns favor surgical intervention, but radiation, immunotherapy, and chemotherapy are also used. We propose that lesions histologically indicative of SCM behave prognostically and are treated similarly to conventional melanoma.
